# Thyroid Eye Disease: Advancements in Orbital and Ocular Pathology Management

**DOI:** 10.3390/jpm14070776

**Published:** 2024-07-22

**Authors:** Anna Scarabosio, Pier Luigi Surico, Rohan Bir Singh, Vlad Tereshenko, Mutali Musa, Fabiana D’Esposito, Andrea Russo, Antonio Longo, Caterina Gagliano, Edoardo Agosti, Etash Jhanji, Marco Zeppieri

**Affiliations:** 1 Clinic of Plastic and Reconstructive Surgery, Ospedale Santa Maria della Misericordia, 33100 Udine, Italy; scarabosioanna@gmail.com; 2 Department of Plastic and Reconstructive Surgery, Mass General Hospital, Harvard Medical School, Boston, MA 02114, USA; 3 Schepens Eye Research Institute of Mass Eye and Ear, Harvard Medical School, Boston, MA 02114, USA; pierluigi.surico@gmail.com (P.L.S.);; 4 Department of Ophthalmology, Campus Bio-Medico University, 00128 Rome, Italy; 5 Department of Optometry, University of Benin, Benin 300238, Nigeria; 6 Africa Eye Laser Centre, Km 7, Benin 300105, Nigeria; 7 Imperial College Ophthalmic Research Group (ICORG) Unit, Imperial College, 153-173 Marylebone Rd., London NW1 5QH, UK; 8 Department of Neurosciences, Reproductive Sciences and Dentistry, University of Naples Federico II, Via Pansini 5, 80131 Napoli, Italy; 9 Department of Ophthalmology, University of Catania, 95123 Catania, Italy; 10 Department of Medicine and Surgery, University of Enna “Kore”, Piazza dell’Università, 94100 Enna, Italy; 11 Eye Clinic, Catania University San Marco Hospital, Viale Carlo Azeglio Ciampi, 95121 Catania, Italy; 12 Division of Neurosurgery, Department of Medical and Surgical Specialties, Radiological Sciences and Public Health, University of Brescia, 25123 Brescia, Italy; 13 Department of Ophthalmology, University of Pittsburg, Pittsburg, PA 15260, USA; 14 Department of Ophthalmology, University Hospital of Udine, 33100 Udine, Italy

**Keywords:** thyroid eye disease, orbital pathologies, proptosis, dry eye, autoimmune, teprotumumab, insulin growth factor

## Abstract

Thyroid Eye Disease (TED) is a debilitating autoimmune condition often associated with thyroid dysfunction, leading to significant ocular and orbital morbidity. This review explores recent advancements in the management of TED, focusing on both medical and surgical innovations. The introduction of Teprotumumab, the first FDA-approved drug specifically for TED, marks a pivotal development in medical therapy. Teprotumumab targets the insulin-like growth factor-1 receptor (IGF-1R), effectively reducing inflammation and tissue remodeling. Clinical trials demonstrate its efficacy in reducing proptosis and improving quality of life, making it a cornerstone in the treatment of active, moderate-to-severe TED. Surgical management remains critical for patients with chronic TED or those unresponsive to medical therapy. Advancements in orbital decompression surgery, including image-guided and minimally invasive techniques, offer improved outcomes and reduced complications. Innovations in eyelid and strabismus surgery enhance functional and cosmetic results, further improving patient satisfaction. The management of TED necessitates a multidisciplinary approach involving endocrinologists, ophthalmologists, oculoplastic surgeons, radiologists, and other specialists. This collaborative strategy ensures comprehensive care, addressing the diverse aspects of TED from thyroid dysfunction to ocular health and psychological well-being. Future directions in TED treatment include emerging pharmacological therapies targeting different aspects of the disease’s pathophysiology and advanced surgical techniques aimed at enhancing precision and safety. This review underscores the importance of a personalized, multidisciplinary approach in managing TED, highlighting current advancements, and exploring potential future innovations to improve patient outcomes and quality of life.

## 1. Introduction

Thyroid Eye Disease (TED), also known as Graves’ orbitopathy, is a complex autoimmune condition associated with thyroid dysfunction, primarily hyperthyroidism [1,2]. TED affects the orbital and periorbital tissues, leading to a range of symptoms including proptosis (bulging eyes), eyelid retraction, diplopia (double vision), and, in severe cases, optic neuropathy, which can threaten vision [3,4]. The condition not only poses significant functional impairments but also leads to substantial cosmetic concerns, impacting patients’ quality of life and psychological well-being [2].

The management of TED is multifaceted, requiring both medical and surgical interventions tailored to the disease’s activity and severity. Recent advancements in both fields have significantly enhanced the therapeutic landscape [5,6,7,8,9,10]. This review aims to explore these advancements, providing a comprehensive overview of current and emerging treatment modalities for TED.

A key focus of this review is the innovative medical therapy Teprotumumab. As the first FDA-approved drug specifically for TED, Teprotumumab represents a groundbreaking advancement. We will delve into its mechanism of action, which involves the inhibition of the insulin-like growth factor-1 receptor (IGF-1R), reducing inflammation and tissue remodeling. The clinical trial evidence supporting Teprotumumab’s efficacy and safety will be summarized, along with guidelines for its use and management of potential side effects [6,7,11]

In addition to medical management, surgical interventions play a crucial role, especially for patients with chronic, stable TED or those who do not respond adequately to medical therapy. We will discuss the latest advancements in surgical techniques, including orbital decompression surgery, eyelid surgery, and strabismus surgery. Innovations such as image-guided surgery and minimally invasive approaches are highlighted for their potential to improve outcomes and reduce complications [5,12,13,14].

A multidisciplinary approach is essential in the management of TED, involving endocrinologists, ophthalmologists, oculoplastic surgeons, radiologists, and other specialists. This collaborative strategy ensures comprehensive care addressing all aspects of the disease. 

Lastly, we will look to the future, exploring potential advancements in both surgical techniques and pharmacological treatments. Emerging therapies and innovative approaches hold promise for further improving the management of TED, offering hope for better control of this challenging condition [10,15,16,17].

The main aim of this review is to provide an in-depth understanding of the current state-of-the-art in TED management, emphasizing the importance of a multidisciplinary approach and highlighting recent and potential future advancements. By doing so, we hope to contribute to improved patient care and outcomes for those suffering from this debilitating disease.

## 2. Materials and Methods

The review utilized PubMed and Reference Citation Analysis (RCA) to gather information. PubMed, managed by the National Library of Medicine, was chosen for its extensive coverage of peer-reviewed biomedical literature. The research employed search terms related to “Thyroid Eye Disease” combined with “diagnosis”, “treatment”, “management”, “pathogenesis”, “immunity”, “antibody”, “clinical manifestations”, “autoimmune”, “therapy”, and other relevant keywords. Boolean operators (AND, OR, NOT) ensured comprehensive and relevant results. Only English articles were considered for accessibility. Titles and abstracts were manually screened, and relevant full texts were reviewed to extract data on clinical features, treatment modalities, outcomes, and associated challenges. Manual reference list searches and citation tracking supplemented the electronic search. This strategy aimed to comprehensively cover all aspects of Thyroid Eye Disease to provide a thorough understanding of current knowledge and advancements.

## 3. Thyroid Eye Disease

### 3.1. Pathophysiology

TED is an autoimmune condition often associated with Graves’ disease [18,19]. However, it can also occur in patients who are euthyroid or hypothyroid. The pathophysiology of TED involves a complex interplay of genetic, environmental, and immunological factors. Central to its development is the activation of auto-reactive T lymphocytes, which target antigens shared by the thyroid gland and orbital tissues. Key antigens implicated in TED include the thyroid-stimulating hormone receptor (TSHR) and the insulin-like growth factor-1 receptor (IGF-1R). When autoantibodies bind to these receptors, they initiate an inflammatory cascade [20,21].

**Figure 1:**
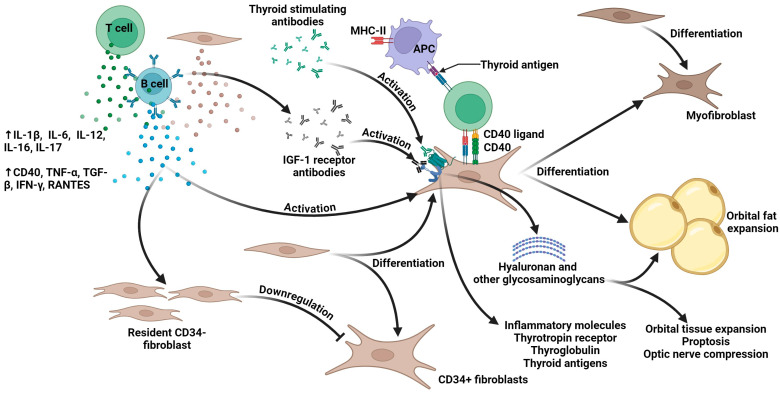
**Pathogenesis of Thyroid Eye Disease (TED).** ↑ (increased secretion); APC (Antigen Presenting Cell); IFN-γ (Interferon gamma); IGF-1 (Insulin-like Growth Factor 1); IL- (Interleukin); MHC-II (Major Histocompatibility Complex class II); RANTES (Regulated on Activation, Normal T Cell Expressed and Secreted); TGF-β (Transforming Growth Factor beta); TNF-α (Tumor Necrosis Factor alpha).

In the early stages of TED, there is significant inflammation and infiltration of the orbital tissues by immune cells, particularly T cells and B cells. These cells release cytokines, leading to the activation of orbital fibroblasts [18,19]. Activated fibroblasts proliferate and differentiate into myofibroblasts and adipocytes, contributing to tissue remodeling. Glycosaminoglycans, particularly hyaluronan, are produced in excess, causing edema and increasing orbital volume. This process results in the characteristic swelling and fibrosis seen in the orbital tissues [18,20,22]. Molecular mechanisms are summarized in Figure 1.

### 3.2. Clinical Manifestations 

TED presents a range of clinical manifestations that vary in severity and can significantly impact the quality of life. These manifestations are generally categorized into two phases: the active (inflammatory) phase and the chronic (fibrotic) phase.

In the active phase, patients often experience symptoms such as orbital pain, redness, and swelling. This phase is marked by inflammation and the acute onset of symptoms. Common clinical features include proptosis, periorbital edema, and conjunctival redness. Patients may also report a sensation of grittiness or discomfort in the eyes, increased tearing, and photophobia [23]. In fact, dry eye disease (DED) is one of the most common features of TED, characterized by distinct and complex etiologies [24]. In TED patients, incomplete blinking and significant loss of Meibomian gland structure in the eyelids are more prominent than in typical DED cases [25]. Initially, DED in TED arises from orbital inflammation, while exposure-related issues contribute to its persistence later [26]. 

Occult TED should be considered in the differential diagnosis of dry eye, presenting with increased corneal fluorescein staining, rapid tear break-up time, and abnormal Schirmer test results, as it can cause inflammatory ocular surface disease with dry eye symptoms [27,28,29]. The exact mechanisms linking TED to DED remain unclear, but it is believed that multiple factors interact synergistically. These include tear film dysfunction due to increased evaporation and/or ocular inflammation, abnormal stimulation of lacrimal glands leading to hyposecretion, and other yet unidentified mechanisms [30]. Effective treatment for DED in TED patients should address both symptom relief and underlying inflammation, utilizing lubricants to alleviate dryness and anti-inflammatory therapies to tackle the root causes [31].

Among with DED, Superior limbal keratitis (SLK) is another typical ocular surface manifestation of TED, contributing significantly to moderate to severe ocular discomfort. SLK is characterized by inflammation of the superior corneal and conjunctival regions, often resulting in redness, pain, photophobia, and a foreign body sensation [32,33]. This condition exacerbates the ocular surface disease commonly seen in TED patients, adding to the overall burden of dry eye symptoms and discomfort. Proper diagnosis and management of SLK are crucial in TED patients to alleviate symptoms and improve quality of life. Treatment often involves a combination of anti-inflammatory therapies and lubricants to address inflammation and surface irregularities. In severe cases, surgical intervention such as conjunctival resection or orbital decompression may be necessary to provide lasting relief and improve ocular surface health [34,35,36,37].

**Figure 2:**
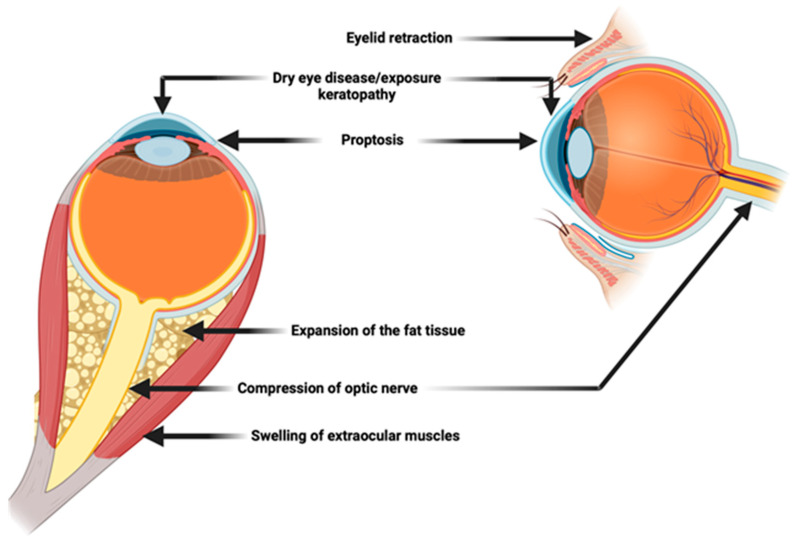
**Thyroid Eye Disease clinical manifestations**.

In the TED spectrum, diplopia (double vision) can occur due to inflammation and subsequent fibrosis of the extraocular muscles, leading to restricted eye movements. As the disease progresses to the chronic phase, inflammation subsides, but fibrosis and tissue remodeling predominate [38]. Proptosis may persist, and lid retraction becomes more apparent. This phase is characterized by less pain but more pronounced mechanical issues, such as exposure keratopathy due to incomplete eyelid closure, and continued diplopia. Severe cases of TED can lead to compressive optic neuropathy, where the optic nerve is compressed by swollen and fibrotic tissues, potentially resulting in vision loss [39,40,41,42]. Clinical manifestations are summarized in Figure 2.

Overall, TED’s clinical course varies widely among patients. Some experience mild symptoms that resolve spontaneously, while others suffer from severe, debilitating manifestations requiring intensive treatment.

The differential diagnosis of TED is critical and involves distinguishing it from other conditions that can present with similar ocular symptoms. Conditions such as dry eye syndrome, conjunctivitis, and orbital cellulitis can mimic TED due to overlapping features like redness, swelling, and irritation of the eyes. Additionally, idiopathic orbital inflammation, myasthenia gravis, and tumors of the orbit can present with proptosis and diplopia, similar to TED.

A comprehensive clinical evaluation is crucial for assessing the severity and activity of TED. This typically includes a detailed history, a thorough ophthalmic examination, and imaging studies such as orbital CT or MRI to evaluate the extent of tissue involvement. The Clinical Activity Score (CAS) is often used to quantify disease activity, guiding therapeutic decisions [8,38].

In summary, TED is a complex, multifaceted disease with significant variability in clinical presentation and progression. Understanding its pathophysiology and clinical manifestations is essential for developing effective management strategies tailored to the individual patient’s needs.

### 3.3. Management

Advancing treatment options for Thyroid Eye Disease (TED) is of paramount importance due to the profound impact this condition has on patients’ quality of life. TED can cause significant functional impairments, including vision loss and severe ocular discomfort, as well as aesthetic disfigurement due to proptosis and lid retraction [4,43,44]. These symptoms can lead to psychological distress and social isolation. Current treatments, while effective for some, often have limitations and may not address all aspects of the disease. Surgical interventions can be invasive with substantial recovery times, and traditional medical therapies may not always provide adequate symptom control [10,45]. According to the 2021 European group guidelines [8], for moderate-to-severe and active TED, intravenous (i.v.) glucocorticoids are more effective and better tolerated than oral glucocorticoids. Current evidence recommends a combination of i.v. methylprednisolone and mycophenolate sodium as first-line treatment. Second-line treatments include a second course of i.v. methylprednisolone, oral prednisone/prednisolone with cyclosporine or azathioprine, orbital radiotherapy with glucocorticoids, teprotumumab, rituximab, and tocilizumab. Sight-threatening TED is treated with high doses of i.v. methylprednisolone and, if necessary, urgent orbital decompression. Rehabilitative surgery is reserved for inactive residual manifestations [8]. Therefore, there is a critical need for innovative treatment approaches that offer improved efficacy, safety, and patient outcomes. Advancements in both surgical techniques and novel pharmacological therapies have the potential to transform the management of TED, providing patients with more comprehensive and less invasive treatment options.

#### 3.3.1. Orbital Decompression Surgery 

Orbital decompression surgery is a cornerstone in the surgical management of TED. The goal of this procedure is to create additional space within the orbit to alleviate proptosis and optic neuropathy by removing bone and/or fat. Several techniques have been described in the last 20 years.

Historically, orbital decompression involved the removal of the orbital walls—primarily the medial and lateral walls. The extent of decompression varied, but these early techniques often led to complications such as new-onset strabismus, sinusitis, and facial asymmetry [46,47].

Contemporary orbital decompression techniques have evolved to minimize complications and improve outcomes. These include lateral wall decompression, which involves removing bone from the lateral wall of the orbit. This provides significant space for orbital contents to expand and is often preferred for its lower risk of inducing strabismus compared to medial wall decompression. This approach can be performed through an external incision or endoscopically through the conjunctiva [47,48].

Medial wall decompression involves removing part of the ethmoid bone, which forms the medial wall of the orbit. While effective for reducing severe proptosis, it carries a higher risk of postoperative diplopia due to the proximity of the medial rectus muscle. A more balanced approach, combining lateral and medial wall decompression, allows for more extensive reduction of proptosis while potentially mitigating the risk of inducing strabismus. The orbital floor can also be decompressed in cases of severe proptosis or optic neuropathy. This involves removing bone from the orbital floor to allow downward displacement of the globe. Modern techniques often use endoscopic approaches to access the orbital floor and medial wall, reducing external scarring and improving precision [47,48].

A recent study investigates the management of dysthyroid optic neuropathy (DON), a severe complication of TED, through three different surgical protocols: modified extended orbital apex decompression, two-wall decompression (inferior and lateral), and three-wall decompression (inferior, lateral, and medial) [49]. The findings indicate that both the extended endonasal approach and three-wall decompression are effective for managing DON. The choice between these methods depends on the degree of proptosis, the patient’s general condition, and the extent of eye surface damage.

Clinical outcomes of orbital decompression surgery have significantly improved with these advanced techniques. Reduction in proptosis is typically substantial, with studies reporting an average reduction of 3–6 mm. Optic neuropathy, if present, is often resolved or significantly improved, reducing the risk of permanent vision loss. Patient satisfaction is generally high, particularly when a multidisciplinary approach is used to customize the surgical plan to the patient’s specific anatomy and disease severity.

Complications, while less common with modern techniques, can still occur. These include new-onset strabismus, sinusitis, and infraorbital nerve hypoesthesia. Careful patient selection and meticulous surgical technique are essential to minimize these risks. Long-term follow-up studies indicate that most patients experience sustained improvements in both function and appearance, contributing to better overall quality of life [46,47,48,50].

#### 3.3.2. Eyelid Surgery 

Eyelid abnormalities, including retraction and lagophthalmos, are common in TED and can cause significant discomfort and exposure keratopathy. Surgical correction of these issues is often necessary to restore function and appearance [3,5].

Upper lid retraction repair traditionally involves recession of the levator palpebrae superioris muscle or Mueller’s muscle. Techniques have evolved to include the use of autologous or alloplastic spacers, such as hard palate grafts or acellular dermal matrix, to provide more predictable and lasting results. Minimally invasive approaches, including the transconjunctival Müller’s muscle recession, offer the advantage of reduced scarring and quicker recovery times. Lower lid retraction repair commonly involves the release of the lower lid retractors and the placement of spacers to support the lid in a more anatomically correct position. Innovations in this area include the use of endoscopic approaches and the introduction of new spacer materials that provide better integration and support [4,43,51].

Blepharoplasty in TED patients is often performed to address the aesthetic and functional issues caused by the disease. Indications include excessive eyelid skin, fat prolapse, and eyelid asymmetry. The techniques used are similar to those in cosmetic blepharoplasty but are tailored to account for the unique challenges posed by TED, such as increased tissue fragility and altered anatomy due to previous inflammation [3,5,8,52].

Outcomes of blepharoplasty in TED patients are generally positive, with high rates of patient satisfaction. Functional improvements, such as better eyelid closure and reduced corneal exposure, are commonly reported. However, careful preoperative planning and patient counseling are essential to manage expectations and ensure that patients understand the potential risks and benefits.

#### 3.3.3. Strabismus Surgery 

Strabismus, or misalignment of the eyes, is a common complication of TED, resulting from fibrosis and restriction of the extraocular muscles. Surgical correction is often necessary to restore binocular vision and relieve diplopia [53].

Strabismus surgery in TED typically involves recessing the affected muscles to reduce the restrictive forces and realign the eyes. The inferior and medial rectus muscles are most affected and are often the targets of surgical intervention. Adjustable suture techniques are frequently used, allowing for postoperative adjustments to fine-tune eye alignment. This approach has been shown to improve outcomes by reducing the risk of overcorrection or undercorrection [54,55].

Recent advances include the use of intraoperative imaging and navigation systems to enhance the precision of muscle placement and the development of less invasive surgical techniques that reduce recovery times and postoperative discomfort.

The success rates of strabismus surgery in TED patients are generally high, with many patients achieving significant improvements in ocular alignment and reduction in diplopia. However, outcomes can be variable, and some patients may require multiple procedures to achieve optimal results. Long-term follow-up studies indicate that most patients maintain good alignment and improved binocular function over time. The impact on ocular motility is also positive, with many patients experiencing increased range of motion and improved control over eye movements [53,54,55,56].

#### 3.3.4. Adjunctive Procedures: Periorbital Fat Management

In addition to primary surgical interventions, several adjunctive procedures can enhance the overall outcomes of TED management.

Orbital fat removal and redistribution are techniques used to address residual proptosis and improve the aesthetic outcomes of TED surgery. Fat removal can be performed through transconjunctival or transcutaneous approaches, depending on the extent of fat prolapse and the surgeon’s preference.

Fat redistribution involves repositioning orbital fat to fill in areas of volume loss or to achieve a more balanced and symmetrical appearance. This technique is particularly useful in patients with significant asymmetry or those who have undergone previous decompression surgery. Advances in this area include the use of microfat grafting and nanofat techniques, which allow for more precise and less invasive fat manipulation [57,58,59].

Overall, these adjunctive procedures can significantly enhance the cosmetic and functional outcomes of TED surgery, providing patients with a more natural and harmonious appearance. The combination of advanced decompression techniques, meticulous eyelid surgery, precise strabismus correction, and thoughtful use of adjunctive procedures represents the current state-of-the-art in surgical management of TED. These advances have collectively improved patient outcomes, reduced complication rates, and increased overall patient satisfaction, contributing to a better quality of life for individuals affected by this challenging condition [59,60,61].

## 4. Teprotumumab in Thyroid Eye Disease 

### 4.1. Mechanism of Action and Efficacy: How Does It Work?

**Figure 3:**
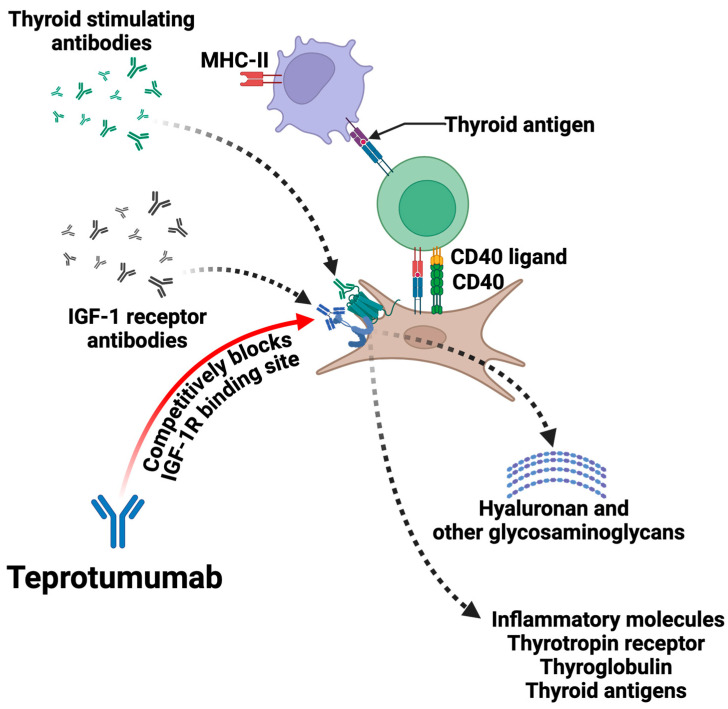
**Teprotumumab mechanism of action.** CD40 (Cluster of Differentiation 40); IGF-1 (Insulin-like Growth Factor 1); IGF-1R (Insulin-like Growth Factor 1 Receptor); MHC-II (Major Histocompatibility Complex class II).

Teprotumumab is a fully human monoclonal antibody that targets the insulin-like growth factor-1 receptor (IGF-1R) (Figure 3). IGF-1R is a critical player in the pathogenesis of TED. In patients with TED, the autoimmune response leads to the activation of IGF-1R, which is overexpressed on the surface of orbital fibroblasts and other cells within the eye orbit. This activation contributes to the inflammatory process and tissue remodeling characteristic of TED [62,63,64,65].

Teprotumumab binds to IGF-1R, inhibiting its activation and downstream signaling pathways. This inhibition reduces the production of pro-inflammatory cytokines and prevents the differentiation of orbital fibroblasts into adipocytes and myofibroblasts. As a result, Teprotumumab diminishes the inflammatory response, reduces tissue expansion and fibrosis, and ultimately alleviates the symptoms of TED, including proptosis and diplopia [7]. 

The efficacy and safety of Teprotumumab were established through two pivotal phase 3 clinical trials: the OPTIC trial and the OPTIC-X extension trial [11,66,67]. These trials provided robust evidence supporting the use of Teprotumumab in the treatment of TED. The OPTIC trial was a randomized, double-blind, placebo-controlled study involving 86 patients with active TED. Participants were randomly assigned to receive either Teprotumumab or a placebo, administered as an intravenous infusion every three weeks for a total of eight infusions. The primary endpoint was a reduction in proptosis of ≥2 mm at week 24. The results of the OPTIC trial were remarkable. Approximately 83% of patients treated with Teprotumumab achieved the primary endpoint, compared to only 10% of those receiving placebo. Additionally, significant improvements were observed in secondary endpoints, including overall response rate, reduction in Clinical Activity Score (CAS), and quality of life measures. The majority of patients experienced substantial reductions in proptosis and improvements in diplopia and functional and emotional well-being [11,62,63,66,67].

The OPTIC-X extension trial provided further evidence of Teprotumumab’s long-term efficacy and safety. This trial enrolled patients who had completed the OPTIC trial and either had a partial response or experienced a recurrence of symptoms. Participants received additional Teprotumumab infusions, resulting in sustained and further improvements in proptosis, CAS, and overall quality of life. The combined data from these trials underscore Teprotumumab’s role as a transformative therapy for TED [67].

### 4.2. Indications and Guidelines for Teprotumumab Use 

Teprotumumab is indicated for the treatment of Thyroid Eye Disease in adult patients. It is particularly beneficial for those with active, moderate-to-severe TED. The recommended dosing regimen involves an initial infusion of 10 mg/kg, followed by subsequent infusions of 20 mg/kg every three weeks for a total of eight infusions [63,64,65].

Before initiating treatment with Teprotumumab, a thorough evaluation of the patient’s disease activity and severity is necessary. Teprotumumab is most effective during the active phase of TED, characterized by ongoing inflammation and rapid progression of symptoms. It is less effective in the chronic, fibrotic stage of the disease when the inflammatory component has subsided [7,11,62,64,65,66].

Patients undergoing treatment with Teprotumumab should be monitored regularly to assess their response to therapy and to manage any potential adverse effects. Baseline assessments should include a detailed ophthalmic examination, including measurements of proptosis, diplopia, and CAS. Subsequent evaluations should occur at each infusion visit to track progress and adjust treatment as necessary.

### 4.3. Potential Adverse Effects and Their Management

Like all medications, Teprotumumab can cause side effects, although many patients tolerate the drug well. The most common adverse effects reported in clinical trials include muscle spasm, nausea, alopecia, diarrhea, fatigue, hyperglycemia, and hearing impairment.

Muscle spasms and cramps are among the most frequently reported side effects. These symptoms are generally mild to moderate in severity and can often be managed with over-the-counter analgesics and muscle relaxants. Ensuring adequate hydration and electrolyte balance may also help alleviate these symptoms. Nausea and diarrhea were also relatively common but typically mild. Patients experiencing these symptoms should be advised to maintain hydration and may use antiemetic or antidiarrheal medications if necessary. Alopecia, or hair thinning, was reported by some patients but was generally mild and reversible upon completion of therapy [68,69,70]. Patients should be reassured that this side effect is typically temporary. Fatigue was another common side effect, often mild to moderate. Encouraging patients to balance activity with rest and to maintain a healthy diet can help manage fatigue. Hyperglycemia, or elevated blood sugar levels, was observed in some patients, particularly those with pre-existing diabetes or other risk factors for glucose intolerance. Blood glucose levels should be monitored regularly in these patients, and adjustments to diabetes medications may be necessary to maintain glycemic control [66,70,71]. 

Hearing impairment, including symptoms such as tinnitus and hypoacusis, was reported in some patients [72]. These symptoms were generally mild to moderate but warrant careful monitoring [73]. Patients should be advised to report any changes in hearing immediately, and audiometric testing may be necessary for those with significant symptoms. In rare cases, patients may experience more severe adverse effects, including infusion reactions and hypersensitivity [74]. Infusion reactions, such as flushing, headache, and shortness of breath, can occur during or shortly after the infusion [11]. Pre-medication with antihistamines and corticosteroids may reduce the risk of these reactions. If a severe hypersensitivity reaction occurs, Teprotumumab should be discontinued immediately, and appropriate medical treatment should be initiated [11,62,68,69,75].

Overall, the management of side effects involves regular monitoring, patient education, and supportive care. Most adverse effects are mild to moderate and manageable with appropriate interventions. The benefits of Teprotumumab in reducing the symptoms and progression of TED generally outweigh the risks, particularly for patients with significant disease activity [68,69,75].

In conclusion, Teprotumumab represents a significant advancement in the medical management of TED. Its targeted mechanism of action, robust clinical trial data, and well-defined safety profile make it a valuable treatment option for patients with active, moderate-to-severe TED. As with any therapy, careful patient selection, regular monitoring, and proactive management of side effects are essential to maximize the therapeutic benefits and ensure patient safety. Teprotumumab offers new hope to patients suffering from TED, providing a much-needed option to improve their vision, appearance, and overall quality of life [9,10].

### 4.4. A Huge Limit: The Cost

The cost of Teprotumumab (teprotumumab) is a significant consideration for patients and healthcare providers. As of 2024, the list price for a single infusion of Teprotumumab is approximately $15,500. Given that the recommended treatment regimen involves an initial infusion followed by seven additional infusions over a 24-week period, the total cost of the complete treatment course can exceed $120,000 [17,76]. These costs do not include additional expenses such as administration fees or the costs associated with managing potential side effects. While the high cost reflects the complexity and novelty of this biologic therapy, it poses a substantial financial burden for many patients. Insurance coverage can vary, and patients may require assistance programs or support from pharmaceutical companies to help mitigate these expenses. Consequently, the cost of Teprotumumab is a critical factor in the broader discussion about access to innovative therapies for Thyroid Eye Disease [17,77,78,79].

## 5. Other Drugs 

In addition to teprotumumab, recent studies have proposed several new pharmacological options for the management of TED. Tocilizumab, a monoclonal antibody targeting the interleukin-6 receptor, has shown promising results in reducing inflammation and improving clinical outcomes in TED. Studies have demonstrated its efficacy in reducing proptosis and improving diplopia and quality of life measures [80,81]. Rituximab, a monoclonal antibody targeting CD20-positive B cells, has been investigated for its immunomodulatory effects in TED, showing potential benefits in reducing disease activity and severity [82,83]. Sirolimus, an mTOR inhibitor, has also been explored for its ability to inhibit T-cell proliferation and reduce inflammation in TED [84]. Mycophenolate sodium, known for its immunosuppressive properties, has been recommended in combination with glucocorticoids as first-line therapy, contributing to the management of active TED [85].Statins, traditionally used for lipid-lowering, have shown anti-inflammatory properties that may benefit patients with TED by modulating immune responses [86]. These treatments offer diverse mechanisms of action and represent evolving options in the pharmacological management of TED, supported by studies highlighting their efficacy and safety profiles.

## 6. Multidisciplinary Approach 

Managing Thyroid Eye Disease (TED) effectively requires a multidisciplinary approach due to the complexity and variability of the disease. This approach ensures that patients receive comprehensive care tailored to their individual needs, which can significantly improve outcomes and quality of life [45].

A multidisciplinary team for TED typically includes endocrinologists, ophthalmologists, oculoplastic surgeons, radiologists, and sometimes rheumatologists and psychologists. Each specialist brings unique expertise that is crucial for addressing the various aspects of TED. Endocrinologists play a key role in managing the underlying thyroid dysfunction that is often associated with TED. Their expertise is crucial for stabilizing thyroid hormone levels, which can help mitigate some of the inflammatory aspects of TED [10,31]. Ophthalmologists, particularly those specializing in orbital diseases, are central to diagnosing and monitoring TED. They conduct detailed eye examinations, assess disease severity, and track progression over time. Oculoplastic surgeons are essential for managing the structural and cosmetic complications of TED. They perform orbital decompression, eyelid surgeries, and strabismus surgeries, which are critical for restoring function and appearance. Radiologists contribute by providing imaging studies such as CT and MRI scans, which help in accurately assessing the extent of orbital involvement and planning surgical interventions [87,88,89]. Rheumatologists may be involved in cases where systemic immunosuppressive therapy is needed, particularly for patients with severe inflammation that does not respond to standard treatments. Psychologists or psychiatrists can provide support for the psychological impact of TED, including body image issues and depression, which are common due to the visible nature of the disease. 

This collaborative approach ensures that all aspects of TED are addressed, from thyroid dysfunction and eye health to psychological well-being, providing a holistic treatment plan that optimizes patient outcomes.

## 7. Patient Selection and Customized Plans: When to Perform Surgery

Selecting the appropriate treatment for TED requires careful consideration of several factors, including disease severity, phase (active vs. chronic), patient symptoms, and overall health. For patients with active, moderate-to-severe TED, medical management with Teprotumumab (teprotumumab) is often the first-line treatment [11,70,71,78]. Teprotumumab has been shown to reduce inflammation, decrease proptosis, and improve quality of life in these patients. Candidates for Teprotumumab typically exhibit significant inflammation and rapidly progressing symptoms. Patients should be in the active phase of TED, where the disease is characterized by active inflammation and swelling. Surgical management is considered for patients who have stable disease or are in the chronic phase, where fibrosis and tissue remodeling predominate. Surgery may also be indicated for those who do not respond to medical therapy or have severe complications such as compressive optic neuropathy [5,14,63,64,65]. 

Orbital decompression surgery is often performed to alleviate severe proptosis and optic neuropathy. Candidates for this surgery usually have significant proptosis causing functional or cosmetic concerns, or evidence of optic nerve compression threatening vision [46,47]. Eyelid surgery is indicated for patients with significant eyelid retraction or lagophthalmos causing exposure keratopathy [35]. Patients with chronic, stable disease who have persistent eyelid abnormalities despite medical management are typical candidates. 

Strabismus surgery is considered for patients with restrictive strabismus and diplopia that interfere with daily activities. This surgery is generally performed after the inflammatory phase has subsided and the disease is stable. Customized treatment plans are developed based on a comprehensive assessment of each patient’s specific needs. This approach ensures that the chosen interventions provide the maximum benefit with the least risk.

## 8. Future Directions and Emerging Therapeutic Strategies

Advances in both surgical and medical management are continuously evolving, offering new hope for TED patients. The future of surgical management for TED includes several promising innovations aimed at improving outcomes and reducing complications [15].

One area of advancement is the use of image-guided surgery, which employs advanced imaging technologies such as intraoperative CT and MRI to provide real-time visualization of the surgical field. This allows for more precise and safer removal of bone and orbital fat, minimizing the risk of complications such as new-onset strabismus or injury to the optic nerve [54,55]. Minimally invasive surgical techniques are also gaining traction. Endoscopic approaches for orbital decompression and eyelid surgery reduce the need for large incisions, leading to quicker recovery times and less postoperative discomfort [90,91]. These techniques also offer improved cosmetic outcomes by minimizing visible scars. Another promising development is the use of customized implants and spacers tailored to the patient’s anatomy [52,92,93,94,95]. These implants can be used in eyelid surgery to provide more predictable and lasting results, reducing the need for revision surgeries [10,45,48,78].

In addition to Teprotumumab, several new pharmacological treatments are under investigation for TED, focusing on targeting different aspects of the disease’s pathophysiology (ongoing clinical trials NCT05015127, NCT05683496, NCT06126783, NCT05987423, NCT06384547, NCT06112340, NCT06088979). IGF-1R antagonists, similar to Teprotumumab, are being explored for their potential to further inhibit the pathways involved in TED. These agents could offer alternative or complementary options for patients who do not respond adequately to Teprotumumab.

Biologic agents targeting other components of the immune system, such as TNF-alpha inhibitors and IL-6 inhibitors, are also being studied. These drugs aim to reduce inflammation more broadly, potentially benefiting patients with severe, refractory TED [10,15,80,96].

Small molecule inhibitors targeting specific intracellular signaling pathways involved in fibroblast activation and tissue remodeling are another area of active research. These drugs could help prevent the progression of fibrosis in chronic TED, improving long-term outcomes [9,75,78].

Gene therapy is an emerging field with potential applications in TED. By targeting the underlying genetic and molecular mechanisms of the disease, gene therapy could offer a more definitive and long-lasting treatment, particularly for patients with severe or recurrent TED.

## 9. Limitations of the Study

This narrative review has several limitations. Firstly, as a narrative review, it lacks the systematic methodology of a PRISMA-guided review, which may introduce selection bias and limit the reproducibility of the findings. While we have focused particularly on new treatments, such as teprotumumab, we have also covered other aspects of therapy, diagnostics, and clinical manifestations. However, the emphasis on newer treatments might have resulted in less comprehensive coverage of traditional therapies. Additionally, the broad scope of discussing both diagnostics and clinical manifestations, while providing a holistic overview, may not delve deeply into each specific area. These factors should be considered when interpreting the conclusions drawn from this review.

## 10. Conclusions

Overall, the future of TED management looks promising, with ongoing research and development likely to yield new treatments that are more effective, safer, and tailored to the individual needs of patients. This multidisciplinary and innovative approach will continue to improve the quality of life for those affected by this challenging disease.

## References

[1] Bahn R.S. (2010). Graves’ Ophthalmopathy. N. Engl. J. Med..

[2] Weiler D.L. (2017). Thyroid Eye Disease: A Review. Clin. Exp. Optom..

[3] Vasanthapuram V.H., Naik M.N. (2022). Lower Eyelid Entropion in Thyroid Eye Disease. Orbit.

[4] Cypen S.G., Conger J.R., Chen L.E., Tao J.P. (2021). Treatment Options for Lower Eyelid Retraction in Thyroid Eye Disease. Int. Ophthalmol. Clin..

[5] Naik M.N., Walvekar P., Vasanthapuram V.H., Shankar L. (2023). Eyelid Surgery in Thyroid Eye Disease. Ophthalmic Plast. Reconstr. Surg..

[6] Rosenblatt T.R., Chiou C.A., Yoon M.K., Lee N.G., Wolkow N., Freitag S.K. (2024). Change in Upper Eyelid Position after Teprotumumab Treatment for Thyroid Eye Disease. Orbit.

[7] Ozzello D.J., Dallalzadeh L.O., Liu C.Y. (2022). Teprotumumab for Chronic Thyroid Eye Disease. Orbit.

[8] Bartalena L., Kahaly G.J., Baldeschi L., Dayan C.M., Eckstein A., Marcocci C., Marinò M., Vaidya B., Wiersinga W.M., Ayvaz G. (2021). The 2021 European Group on Graves’ Orbitopathy (EUGOGO) Clinical Practice Guidelines for the Medical Management of Graves’ Orbitopathy. Eur. J. Endocrinol..

[9] Hoang T.D., Stocker D.J., Chou E.L., Burch H.B. (2022). 2022 Update on Clinical Management of Graves Disease and Thyroid Eye Disease. Endocrinol. Metab. Clin. N. Am..

[10] Pouso-Diz J.M., Abalo-Lojo J.M., Gonzalez F. (2020). Thyroid Eye Disease: Current and Potential Medical Management. Int. Ophthalmol..

[11] Kahaly G.J., Douglas R.S., Holt R.J., Sile S., Smith T.J. (2021). Teprotumumab for Patients with Active Thyroid Eye Disease: A Pooled Data Analysis, Subgroup Analyses, and off-Treatment Follow-up Results from Two Randomised, Double-Masked, Placebo-Controlled, Multicentre Trials. Lancet Diabetes Endocrinol..

[12] Tran L., Klainguti G., Hoeckele N., Kaeser P.F. (2023). Torsional Strabismus and Vertical Rectus Muscle Surgery in Thyroid Eye Disease. J. Fr. Ophtalmol..

[13] Bernardini F.P., Skippen B., Zambelli A., Riesco B., Devoto M.H. (2018). Simultaneous Aesthetic Eyelid Surgery and Orbital Decompression for Rehabilitation of Thyroid Eye Disease: The One-Stage Approach. Aesthet. Surg. J..

[14] Araya J., Sabharwal S., Briceño C.A. (2023). Surgery in Thyroid Eye Disease. Int. Ophthalmol. Clin..

[15] Barbesino G., Salvi M., Freitag S.K. (2022). Future Projections in Thyroid Eye Disease. J. Clin. Endocrinol. Metab..

[16] Moledina M., Damato E.M., Lee V. (2024). The Changing Landscape of Thyroid Eye Disease: Current Clinical Advances and Future Outlook. Eye.

[17] Perros P., Hegedüs L. (2023). Teprotumumab in Thyroid Eye Disease: Wonder Drug or Great Divider?. Eur. Thyroid. J..

[18] Spadaro J.Z., Kohli A.A. (2023). Pathogenesis of Thyroid Eye Disease. Int. Ophthalmol. Clin..

[19] Smith T.J. (2022). Understanding Pathogenesis Intersects With Effective Treatment for Thyroid Eye Disease. J. Clin. Endocrinol. Metab..

[20] Khong J.J., McNab A.A., Ebeling P.R., Craig J.E., Selva D. (2016). Pathogenesis of Thyroid Eye Disease: Review and Update on Molecular Mechanisms. Br. J. Ophthalmol..

[21] Wall J.R., Lahooti H. (2010). Pathogenesis of Thyroid Eye Disease—Does Autoimmunity against the TSH Receptor Explain All Cases?. Endokrynol. Pol..

[22] Shu X., Shao Y., Chen Y., Zeng C., Huang X., Wei R. (2024). Immune Checkpoints: New Insights into the Pathogenesis of Thyroid Eye Disease. Front. Immunol..

[23] Smith T.J., Hegedüs L., Lesser I., Perros P., Dorris K., Kinrade M., Troy-Ott P., Wuerth L., Nori M. (2023). How Patients Experience Thyroid Eye Disease. Front. Endocrinol..

[24] Qian L., Wei W. (2022). Identified Risk Factors for Dry Eye Syndrome: A Systematic Review and Meta-Analysis. PLoS ONE.

[25] Park J., Baek S. (2019). Dry Eye Syndrome in Thyroid Eye Disease Patients: The Role of Increased Incomplete Blinking and Meibomian Gland Loss. Acta Ophthalmol..

[26] Lo C., Yang M., Rootman D. (2021). Natural History of Inflammatory and Non-Inflammatory Dry Eye in Thyroid Eye Disease. Orbit.

[27] Gupta A., Sadeghi P.B., Akpek E.K. (2009). Occult Thyroid Eye Disease in Patients Presenting with Dry Eye Symptoms. Am. J. Ophthalmol..

[28] Yu K., Bunya V., Maguire M., Asbell P., Ying G.S. (2021). Systemic Conditions Associated with Severity of Dry Eye Signs and Symptoms in the Dry Eye Assessment and Management Study. Ophthalmology.

[29] Liao X., Lai K.K.H., Aljufairi F.M.A.A., Chen W., Hu Z., Wong H.Y.M., Jia R., Wei Y., Tham C.C.Y., Pang C.P. (2023). Ocular Surface Changes in Treatment-Naive Thyroid Eye Disease. J. Clin. Med..

[30] Rana H.S., Akella S.S., Clabeaux C.E., Skurski Z.P., Aakalu V.K. (2022). Ocular Surface Disease in Thyroid Eye Disease: A Narrative Review. Ocul. Surf..

[31] Jones L., Downie L.E., Korb D., Benitez-del-Castillo J.M., Dana R., Deng S.X., Dong P.N., Geerling G., Hida R.Y., Liu Y. (2017). TFOS DEWS II Management and Therapy Report. Ocul. Surf..

[32] Lahoti S., Weiss M., Johnson D.A., Kheirkhah A. (2022). Superior Limbic Keratoconjunctivitis: A Comprehensive Review. Surv. Ophthalmol..

[33] Bron A.J., de Paiva C.S., Chauhan S.K., Bonini S., Gabison E.E., Jain S., Knop E., Markoulli M., Ogawa Y., Perez V. (2017). TFOS DEWS II Pathophysiology Report. Ocul. Surf..

[34] Takahashi Y., Vaidya A., Kakizaki H. (2021). Changes in Eyelid Pressure and Dry Eye Status after Orbital Decompression in Thyroid Eye Disease. J. Clin. Med..

[35] Takahashi Y., Ichinose A., Kakizaki H. (2014). Topical Rebamipide Treatment for Superior Limbic Keratoconjunctivitis in Patients with Thyroid Eye Disease. Am. J. Ophthalmol..

[36] Passons G.A., Wood T.O. (1984). Conjunctival Resection for Superior Limbic Keratoconjunctivitis. Ophthalmology.

[37] Lee D.H., Margolis M.S., Iovieno A., Ling J., Ng T., Djalilian A.R., Yeung S.N. (2023). Superior Limbic Keratoconjunctivitis: Update on Pathophysiology and Management. Ocul. Surf..

[38] Mourits M.P., Prummel M.F., Wiersinga W.M., Koornneef L. (1997). Clinical Activity Score as a Guide in the Management of Patients with Graves’ Ophthalmopathy. Clin. Endocrinol..

[39] Ugradar S., Rootman D.B. (2019). Noninflammatory Thyroid Eye Disease. Ophthalmic Plast. Reconstr. Surg..

[40] Dolman P.J. (2018). Grading Severity and Activity in Thyroid Eye Disease. Ophthalmic Plast. Reconstr. Surg..

[41] Dutton J.J. (2018). Anatomic Considerations in Thyroid Eye Disease. Ophthalmic Plast. Reconstr. Surg..

[42] Johnson B.T., Jameyfield E., Aakalu V.K. (2021). Optic Neuropathy and Diplopia from Thyroid Eye Disease: Update on Pathophysiology and Treatment. Curr. Opin. Neurol..

[43] Lee D.C., Young S.M., Kim Y.D., Woo K.I. (2020). Course of Upper Eyelid Retraction in Thyroid Eye Disease. Br. J. Ophthalmol..

[44] Grisolia A.B.D., Couso R.C., Matayoshi S., Douglas R.S., Briceño C.A. (2017). Non-Surgical Treatment for Eyelid Retraction in Thyroid Eye Disease (TED). Br. J. Ophthalmol..

[45] Jain A.P., Jaru-Ampornpan P., Douglas R.S. (2021). Thyroid Eye Disease: Redefining Its Management—A Review. Clin. Exp. Ophthalmol..

[46] Rootman D.B. (2018). Orbital Decompression for Thyroid Eye Disease. Surv. Ophthalmol..

[47] Jefferis J.M., Jones R.K., Currie Z.I., Tan J.H., Salvi S.M. (2018). Orbital Decompression for Thyroid Eye Disease: Methods, Outcomes, and Complications. Eye.

[48] Pham T.A., Simmons B., Potter N.J., Al-Qurayshi Z., Carter K.D., Graham S.M. (2022). Revision Orbital Decompression for Thyroid Eye Disease. Am. J. Otolaryngol.—Head. Neck Med. Surg..

[49] Dallan I., Cristofani-Mencacci L., Fiacchini G., Benettini G., Picariello M., Lanzolla G., Lazzerini F., Rocchi R., Turri-Zanoni M., Menconi F. (2022). Functional Outcomes and Complications in Refractory Dysthyroid Optic Neuropathy Management: Experience with 3 Different Surgical Protocols. Am. J. Otolaryngol..

[50] Juniat V., Abbeel L., Anthony McGilligan J., Curragh D., Selva D., Rajak S. (2019). Endoscopic Orbital Decompression by Oculoplastic Surgeons for Proptosis in Thyroid Eye Disease. Ophthalmic Plast. Reconstr. Surg..

[51] Korn B.S., Kikkawa D.O., Cohen S.R., Hartstein M., Annunziata C.C. (2008). Treatment of Lower Eyelid Malposition with Dermis Fat Grafting. Ophthalmology.

[52] Osaki T.H., Monteiro L.G., Osaki M.H. (2022). Management of Eyelid Retraction Related to Thyroid Eye Disease. Taiwan. J. Ophthalmol..

[53] Boulakh L., Nygaard B., Bek T., Faber J., Heegaard S., Toft P.B., Poulsen H.E., Toft-Petersen A.P., Hesgaard H.B., Ellervik C. (2022). Nationwide Incidence of Thyroid Eye Disease and Cumulative Incidence of Strabismus and Surgical Interventions in Denmark. JAMA Ophthalmol..

[54] Harrad R. (2015). Management of Strabismus in Thyroid Eye Disease. Eye.

[55] Akbari M.R., Mirmohammadsadeghi A., Mahmoudzadeh R., Veisi A. (2020). Management of Thyroid Eye Disease-Related Strabismus. J. Curr. Ophthalmol..

[56] Hwang B., Heo H., Lambert S.R. (2022). Risk Factors for Reoperation after Strabismus Surgery among Patients with Thyroid Eye Disease. Am. J. Ophthalmol..

[57] Doumit G., Abouhassan W., Yaremchuk M.J. (2014). Aesthetic Refinements in the Treatment of Graves Ophthalmopathy. Plast. Reconstr. Surg..

[58] Choudhary M.M., Zhang K.R., Johnson S., Hwang C.J., Chon B.H., Perry J.D. (2020). Temporal Fat Pad Volume in Patients With Thyroid Eye Disease. Ophthalmic Plast. Reconstr. Surg..

[59] Roncevic R. (2008). Correction of Exophthalmos and Eyelid Deformities in Patients with Severe Thyroid Ophthalmopathy. J. Craniofacial Surg..

[60] Valencia M.R.P., Miyazaki H., Kakizaki H., Takahashi Y. (2020). Thickness of Retro- and Sub-Orbicularis Oculi Fat in Thyroid Eye Disease: Comparison With Controls and Its Influential Factors. Ophthalmic Plast. Reconstr. Surg..

[61] Rončević R., Rončević D. (1995). Surgical Treatment of Severe Dysthyroid Ophthalmopathy—Long-Term Results. J. Cranio-Maxillofac. Surg..

[62] Smith T.J., Kahaly G.J., Ezra D.G., Fleming J.C., Dailey R.A., Tang R.A., Harris G.J., Antonelli A., Salvi M., Goldberg R.A. (2017). Teprotumumab for Thyroid-Associated Ophthalmopathy. N. Engl. J. Med..

[63] Douglas R.S., Couch S., Wester S.T., Fowler B.T., Liu C.Y., Subramanian P.S., Tang R., Nguyen Q.T., Maamari R.N., Ugradar S. (2024). Efficacy and Safety of Teprotumumab in Patients With Thyroid Eye Disease of Long Duration and Low Disease Activity. J. Clin. Endocrinol. Metab..

[64] Ding Y., Yang S., Gao H. (2021). Teprotumumab: The Dawn of Therapies in Moderate-to-Severe Thyroid-Associated Ophthalmopathy. Horm. Metab. Res..

[65] Yu C.Y., Keen J.A., Shriver E.M. (2022). Teprotumumab. Adv. Ophthalmol. Optom..

[66] Douglas R.S., Kahaly G.J., Patel A., Sile S., Thompson E.H.Z., Perdok R., Fleming J.C., Fowler B.T., Marcocci C., Marinò M. (2020). Teprotumumab for the Treatment of Active Thyroid Eye Disease. N. Engl. J. Med..

[67] Douglas R.S., Kahaly G.J., Ugradar S., Elflein H., Ponto K.A., Fowler B.T., Dailey R., Harris G.J., Schiffman J., Tang R. (2022). Teprotumumab Efficacy, Safety, and Durability in Longer-Duration Thyroid Eye Disease and Re-Treatment: OPTIC-X Study. Ophthalmology.

[68] Toro-Tobon D., Rachmasari K.N., Bradley E.A., Wagner L.H., Tooley A.A., Stokken J.K., Stan M.N. (2023). Medical Therapy in Patients with Moderate to Severe, Steroid-Resistant, Thyroid Eye Disease. Thyroid..

[69] Stan M.N., Krieger C.C. (2023). The Adverse Effects Profile of Teprotumumab. J. Clin. Endocrinol. Metab..

[70] Hubschman S., Sojitra B., Ghiam S., Sears C., Hwangbo N., Goldberg R.A., Rootman D.B. (2024). Teprotumumab and Orbital Decompression for the Management of Proptosis in Patients With Thyroid Eye Disease. Ophthalmic Plast. Reconstr. Surg..

[71] Slentz D.H., Nelson C.C., Smith T.J. (2020). Teprotumumab: A Novel Therapeutic Monoclonal Antibody for Thyroid-Associated Ophthalmopathy. Expert. Opin. Investig. Drugs.

[72] Sears C.M., Azad A.D., Amarikwa L., Pham B.H., Men C.J., Kaplan D.N., Liu J., Hoffman A.R., Swanson A., Alyono J. (2022). Hearing Dysfunction After Treatment With Teprotumumab for Thyroid Eye Disease. Am. J. Ophthalmol..

[73] Keen J.A., Correa T., Pham C., Claussen A.D., Hansen M.R., Carter K.D., Shriver E.M. (2024). Frequency and Patterns of Hearing Dysfunction in Patients Treated with Teprotumumab. Ophthalmology.

[74] Dallalzadeh L.O., Ting M., Topilow N., Robbins S.L., Liu C.Y., Burkat C.N., Korn B.S., Kikkawa D.O. (2023). Teprotumumab-Related Cutaneous Hypersensitivity Reactions. Ophthalmic Plast. Reconstr. Surg..

[75] Ugradar S., Kossler A.L., Douglas R., Cockerham K. (2022). A Paradigm Shift in the Management of Thyroid Eye Disease How Teprotumumab Has Changed the Therapeutic Interface. J. Neuro-Ophthalmol..

[76] Allen R.C., Bradley E.A., Fante R.G., Lucarelli M.J. (2021). A Perspective on the Current Role of Teprotumumab in Treatment of Thyroid Eye Disease. Ophthalmology.

[77] Xavier N.F., Lucena D.T., Cruz A.A.V. (2023). Monoclonal Antibodies for the Treatment of Graves Orbitopathy: Precision Medicine?. Ophthalmic Plast. Reconstr. Surg..

[78] Mishra S., Maurya V.K., Kumar S., Ankita, Kaur A., Saxena S.K. (2020). Clinical Management and Therapeutic Strategies for the Thyroid-Associated Ophthalmopathy: Current and Future Perspectives. Curr. Eye Res..

[79] Gupta V., Hammond C.L., Roztocil E., Gonzalez M.O., Feldon S.E., Woeller C.F. (2022). Thinking inside the Box: Current Insights into Targeting Orbital Tissue Remodeling and Inflammation in Thyroid Eye Disease. Surv. Ophthalmol..

[80] Pampín-Sánchez R., Martínez-Mugica-Barbosa C., Fonseca-Aizpuru E.M., Barbazán-Vázquez F.J., Fernández-González B., Buznego-Súárez L. (2023). Outcome of Tocilizumab Treatment in Corticosteroid-Resistant Thyroid Eye Disease. Med. Clin..

[81] Strianese D. (2018). Efficacy and Safety of Immunosuppressive Agents for Thyroid Eye Disease. Ophthalmic Plast. Reconstr. Surg..

[82] Insull E.A., Sipkova Z., David J., Turner H.E., Norris J.H. (2019). Early Low-Dose Rituximab for Active Thyroid Eye Disease: An Effective and Well-Tolerated Treatment. Clin. Endocrinol..

[83] Savino G., Mandarà E., Gari M., Battendieri R., Corsello S.M., Pontecorvi A. (2015). Intraorbital Injection of Rituximab versus High Dose of Systemic Glucocorticoids in the Treatment of Thyroid-Associated Orbitopathy. Endocrine.

[84] Lanzolla G., Maglionico M.N., Comi S., Menconi F., Piaggi P., Posarelli C., Figus M., Marcocci C., Marinò M. (2022). Sirolimus as a Second-Line Treatment for Graves’ Orbitopathy. J. Endocrinol. Investig..

[85] Kahaly G.J., Riedl M., König J., Pitz S., Ponto K., Diana T., Kampmann E., Kolbe E., Eckstein A., Moeller L.C. (2018). Mycophenolate plus Methylprednisolone versus Methylprednisolone Alone in Active, Moderate-to-Severe Graves’ Orbitopathy (MINGO): A Randomised, Observer-Masked, Multicentre Trial. Lancet Diabetes Endocrinol..

[86] Lanzolla G., Sabini E., Leo M., Menconi F., Rocchi R., Sframeli A., Piaggi P., Nardi M., Marcocci C., Marinò M. (2021). Statins for Graves’ Orbitopathy (STAGO): A Phase 2, Open-Label, Adaptive, Single Centre, Randomised Clinical Trial. Lancet Diabetes Endocrinol..

[87] Chaganti S., Mundy K., DeLisi M.P., Nelson K.M., Harrigan R.L., Galloway R.L., Landman B.A., Mawn L.A. (2019). Assessment of Orbital Computed Tomography (CT) Imaging Biomarkers in Patients with Thyroid Eye Disease. J. Digit. Imaging.

[88] Rana K., Juniat V., Patel S., Selva D. (2022). Extraocular Muscle Enlargement. Graefe’s Arch. Clin. Exp. Ophthalmol..

[89] Tran C., Pham C.M., Simmons B.A., Warner L.L., Fuhrmeister L.J., Shriver E.M. (2022). Echographic Assessment of Extraocular Muscle Response to Teprotumumab. Ophthalmic Plast. Reconstr. Surg..

[90] Lima W.T.A., Perches M., Valera F.C.P., Demarco R.C. (2006). Orbital Endoscopic Decompression in Graves Ophthalmopathy. Braz. J. Otorhinolaryngol..

[91] Tu Y., Wu S., Pan Z., Hu X., Zhou G., Shi J., Xu M., Liu W., Wu W. (2022). Endoscopic Transconjunctival Deep Lateral Wall Decompression for Thyroid-Associated Orbitopathy: A Minimally Invasive Alternative: Transconjunctival Endoscopic with Wall Decompression for TAO. Am. J. Ophthalmol..

[92] Park E., Lewis K., Alghoul M.S. (2017). Comparison of Efficacy and Complications Among Various Spacer Grafts in the Treatment of Lower Eyelid Retraction: A Systematic Review. Aesthet. Surg. J..

[93] Liao S.L., Wei Y.H. (2013). Correction of Lower Lid Retraction Using TarSys Bioengineered Grafts for Graves Ophthalmopathy. Am. J. Ophthalmol..

[94] Kim K.Y., Woo Y.J., Jang S.Y., Lee E.J., Yoon J.S. (2017). Correction of Lower Eyelid Retraction Using Acellular Human Dermis During Orbital Decompression. Ophthalmic Plast. Reconstr. Surg..

[95] Tao J.P., Aakalu V.K., Wladis E.J., Sobel R.K., Freitag S.K., Foster J.A., Yen M.T. (2020). Bioengineered Acellular Dermal Matrix Spacer Grafts for Lower Eyelid Retraction Repair: A Report by the American Academy of Ophthalmology. Ophthalmology.

[96] Ayabe R., Rootman D.B., Hwang C.J., Ben-Artzi A., Goldberg R. (2014). Adalimumab as Steroid-Sparing Treatment of Inflammatory-Stage Thyroid Eye Disease. Ophthalmic Plast. Reconstr. Surg..

